# Supplementary material for: TonEBP/NFAT5 promotes obesity and insulin resistance by epigenetic suppression of white adipose tissue beiging
Source: Nat Commun. 2019 Aug 6;10:3536. doi: 10.1038/s41467-019-11302-w (PMC6684655; doi:10.1038/s41467-019-11302-w)
Supplement: Supplementary file 1 — Supplementary Information [file 41467_2019_11302_MOESM1_ESM.pdf]

Supplementary Information

TonEBP/NFAT5 promotes obesity and insulin resistance by epigenetic suppression of white adipose tissue beiging

Lee *et al.*

Supplementary Table 1. Primers used for real time PCR

Species	Gene	Forward primer (5'-3')	Reverse primer (5'-3')
Mouse	*36B4*	TGGCCAATAAGGTGCCAGCTGCTG	CTTGTCTCCAGTCTTTATCAGCTGCAC
	Acadm	AGGATGACGGAGCAGCCAATGA	GCCGTTGATAACATACTCGTCAC
	ACC1	CTGACGTATACTGAACTGGTGTTGGATG	TTTCCAGGCTACCATGCCAATCTC
	Acox1	GCCATTCGATACAGTGCTGTGAG	CCGAGAAAGTGGAAGGCATAGG
	Adiponectin	TGGAATGACAGGAGCTGAAGG	ACACTGAACGCTGAGCGATACACA
	Adrb3	AGGCACAGGAATGCCACTCCAA	GCTTAGCCACAACGAACACTCG
	CD137	CCAAGTACCTTCTCCAGCATAGG	GCGTTGTGGGTAGAGGAGCAAA
	CD36	GAACCACTGCTTTCAAAAACTGG	TGCTGTTCTTTGCCACGTCA
	Cidea	GGTGGACACAGAGGAGTTCTTTC	CGAAGGTGACTCTGGCTATTCC
	CPT1α	GGCATAAACGCAGAGCATTCCTG	CAGTGTCCATCCTCTGAGTAGC
	CyclophilinA	CTGCTGTCTTTGGAACTTTGTCTG	CAGCCATGGTCAACCCCACCG
	Dio2	GGTGGTCAACTTTGGTTCAGCC	AAGTCAGCCACCGAGGAGAACT
	F4/80	CTTTGGCTATGGGCTTCCAGTC	GCAAGGAGGACAGAGTTTATCGTG
	Fasn	CCAGACAGAGAAGAGCCATGGAGG	CCAATGAGGTTGGCCCAGAACTCC
	HSL	GCTGGGCTGTCAAGCACTGT	GTAACTGGGTAGGCTGCCAT
	Leptin	GGGCTTCACCCCATTCTGA	TGGCTATCTGCAGCACATTTTG
	Lipe	GCTCATCTCCTATGACCTACGG	TCCGTGGATGTGAACAACCAGG
	LPL	GCGTAGCAGGAAGTCTGACCAA	AGCGTCATCAGGAGAAAGGCGA
	PGC1α	GAGAATGAGGCAAACTTGCTAGCG	TGCATGGTTCTGAGTGCTAAGACC
	PPARα	AAGACTACCTGCTACCGAAATG	AACATTGGGCCGGTTAAGA
	PPARγ	TTCGCTGATGCACTGCCTATGA	AAGGAATGCGAGTGGTCTTCCA
	PPARγ*2*	TCTTAACTGCCGGATCCACAA	GCCCAAACCTGATGGCATT
	Scd1	TTCTTGCGATACACTCTGGTGC	CGGGATTGAATGTTCTTGTCGT
	Slc27a1	TGCCACAGATCGGCGAGTTCTA	AGTGGCTCCATCGTGTCCTCAT
	Slc2a4	GGTGTGGTCAATACGGTCTTCAC	AGCAGAGCCACGGTCATCAAGA
	SREBP1c	GCCGTGGTGAGAAGCGCACAGCCC	CAAGACAGCAGATTTATTCAGCTTTGC
	TMEM26	TGGTCCTGGATACAGTGCTCAC	GGTGCATTTCAAGAAGCCACAGG
	TNFα	TGGGACAGTGACCTGGACTGT	TTCGGAAAGCCCATTTGAGT
	TonEBP	AAGCAGCCACCACCAAACATGA	AAATTGCATGGGCTGCTGCT
	UCP-1	GTGAACCCGACAACTTCCGAA	TGAAACTCCGGCTGAGAAGAT
Human	*36B4*	TGGTCATCCAGCAGGTGTTCGA	ACAGACACTGGCAACATTGCGG
	Adrb3	ATTGCTTGGGTTGGTCAAATG	AAGGTGAGTGGGAAGGTAGA
	CyclophilinA	TTCATCTGCACTGCCAAGAC	TCGAGTTGTCCACAGTCAGC
	TonEBP	AGCTGTTGTTGCTGCTGATGCT	TCCACTTGCATAGCCTTGCTGT

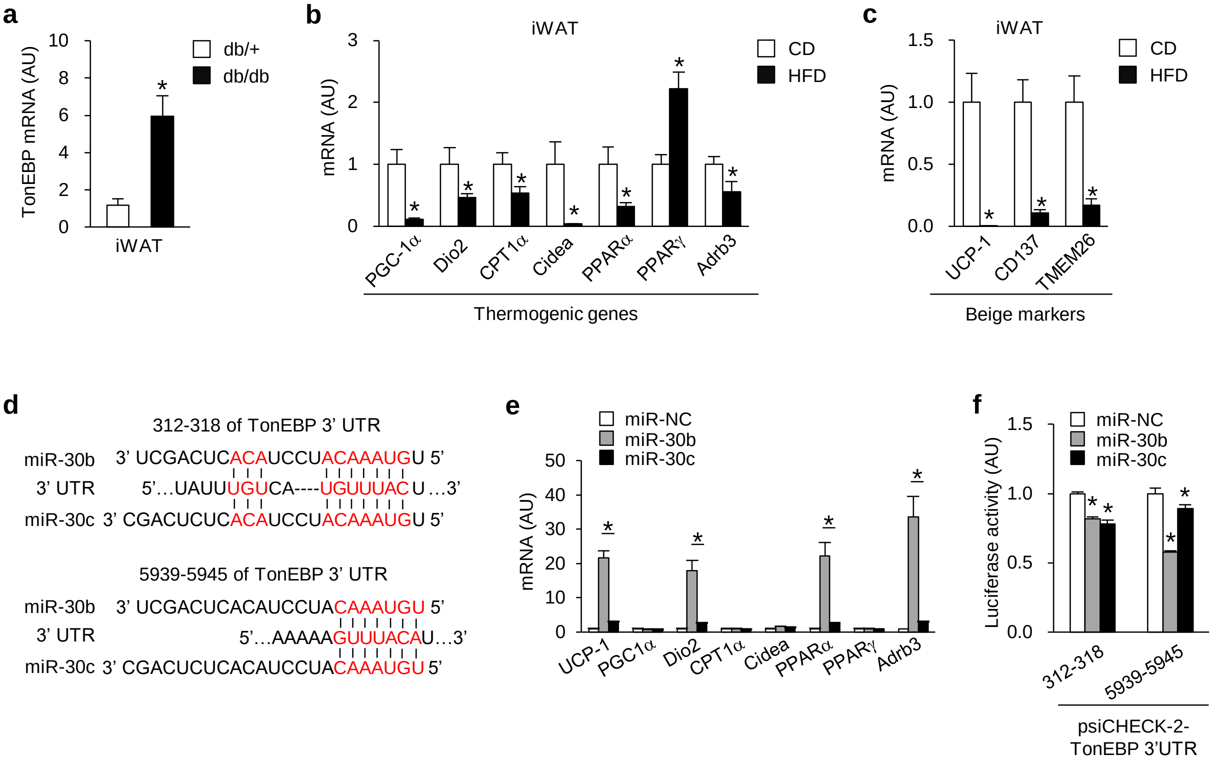

Supplementary Figure 1. Effects of diet and miR on TonEBP, and thermogenic and beige marker genes in adipose tissues and adipocytes.

a TonEBP expression in db/db mice. *TonEBP* mRNA levels in iWAT from 10 weeks old +/db or db/db mice on C57BL/6J background (+/db, n = 5; db/db, n = 7). Data are representative of two independent experiments and presented as mean + s.e.m. * $p < 0.05$ vs. db/+. **b, c** Thermogenic **(b)** and beige marker **(c)** gene expression in iWAT. mRNA levels in iWAT from C57BL/6J mice fed a CD (chow diet, n = 5) or HFD (high fat diet, n = 7) for 16 weeks. All data are representative of four independent experiments and presented as mean + s.e.m. * $p < 0.05$ vs. CD. **d - f** miR30-b and miR30-c promotes thermogenic gene expression. **(d)** Potential sites of miR-30b and miR-30c hybridization on TonEBP 3'-UTR. **(e)** Thermogenic gene mRNA levels in 3T3-L1 adipocytes transfected with miR-negative control (NC), miR-30b or miR-30c (n = 4). **(f)** HEK293 cells were transfected miR-NC, miR-30b or miR-30c followed by transfection of a reporter construct (312-318 or 5939-5945) containing 3′-UTR of TonEBP with putative miR-30b and miR-30c–binding sites (n = 4). All data are representative of three independent experiments and presented as mean + s.d. * p < 0.05 vs. miR-NC. AU, arbitrary unit

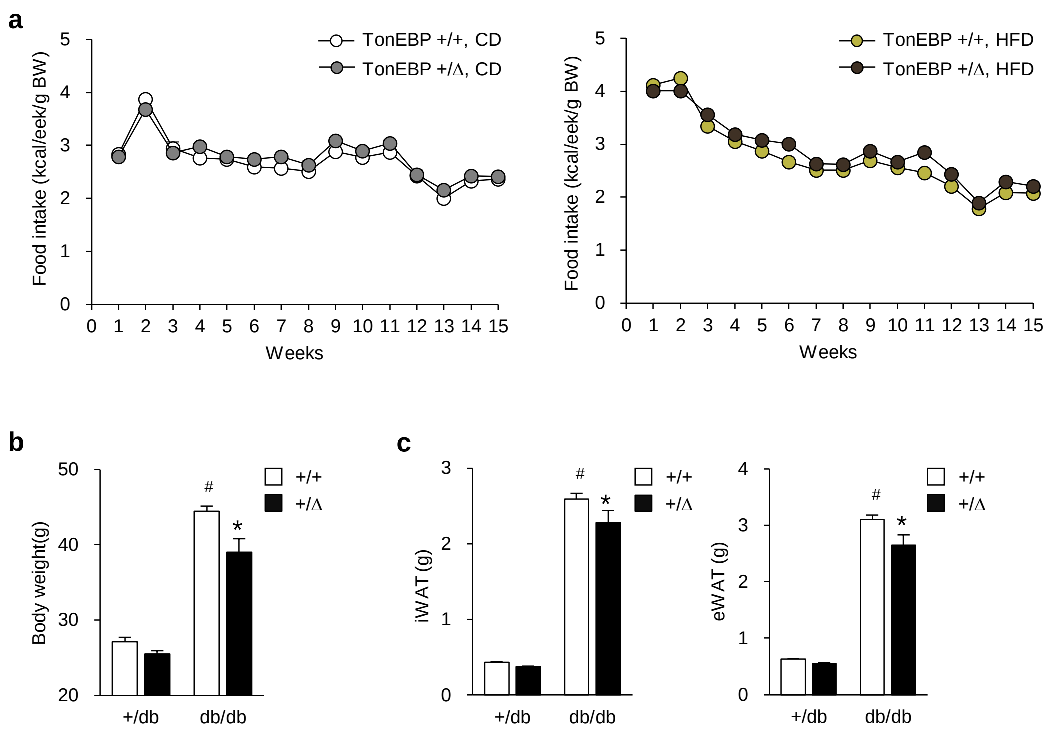

Supplementary Figure 2. Effects of TonEBP haplo-deficiency on food intake and obesity.

a Food intake was not affected by TonEBP haplo-deficiency. Food intake was measured in animals fed with CD (n = 5) or HFD (n = 7). All data are representative of four independent experiments and presented as mean + s.e.m. * $p < 0.05$ vs. +/+. Values of s.e.m. were all smaller than the size of circles for mean values. **b, c** TonEBP haplo-deficiency decreases whole body and fat pad weights in db/db mice (TonEBP +/+, n = 7; TonEBP +/Δ, n = 10). **(b)** Body weight of TonEBP haplo-insufficient (*TonEBP +/Δ, n* = 7) mice on +/db or db/db background and their WT (*TonEBP +/+, n* = 10) littermates. **(c)** Weight of iWAT and eWAT. All data are representative of two independent experiments and presented as mean + s.e.m. # $p < 0.05$ vs. +/db and * $p < 0.05$ vs. TonEBP +/+.

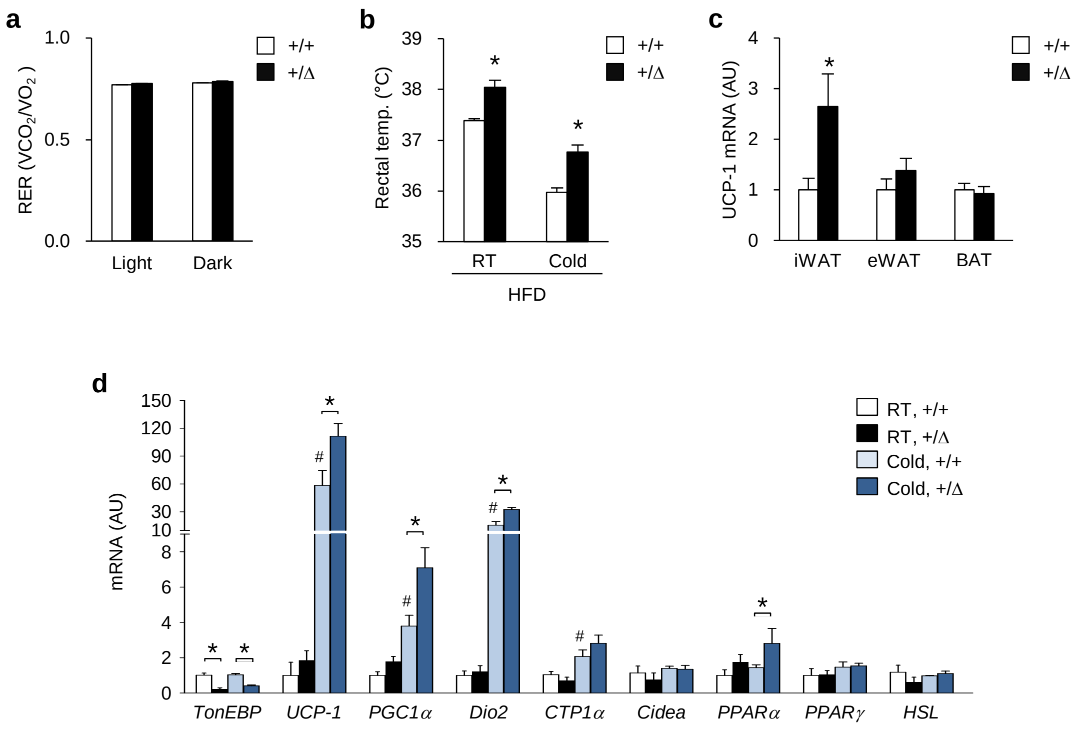

Supplementary Figure 3. Effects of TonEBP haplo-deficiency on thermogenesis and beiging. **a** RER was not affected by TonEBP haplo-deficiency. HFD-fed animals were analyzed by indirect calorimetry to obtain RER (VCO_2/VO_2) (n = 4). Data are representative of three independent experiments. **b** Higher rectal temperature and resistance to cold in TonEBP haplo-deficiency. Rectal temperature (temp.) measured in HFD-fed animals at room temperature (RT) and after exposure to 4°C (Cold) for 6 h (n = 5). **c** Elevated iWAT *UCP-1* mRNA expression in TonEBP haplo-deficiency. *UCP-1* mRNA levels in iWAT, eWAT and BAT from WT (+/+) and TonEBP haplo-deficient mice (+/Δ) (n = 8). **d** Elevated expression of thermogenic genes in TonEBP haplo-deficiency. mRNA abundance of thermogenic genes in iWAT of CD-fed animals exposed to RT or 4°C (cold) (n = 10). Data are presented as mean + s.e.m. # $p < 0.05$ vs. RT **(d)**, * $p < 0.05$ vs. +/+ **(b-d)**. **(a-c)** An unpaired t-test was used for comparisons between two conditions. **(d)** A 1-way ANOVA was used for comparisons between more than two conditions. Tukey's post-hoc test was used for multiple comparisons. AU, arbitrary unit.

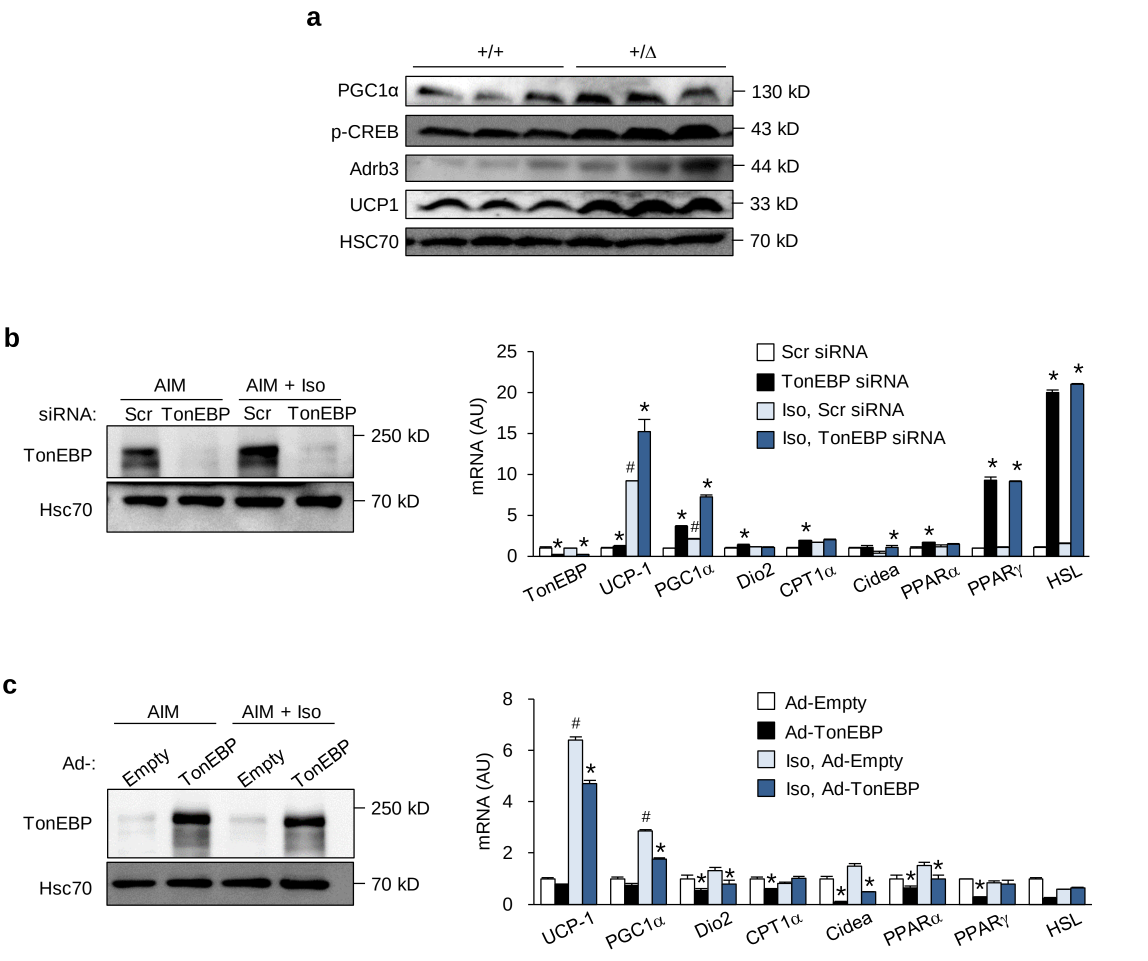

Supplementary Figure 4. Role of TonEBP on thermogenesis and beiging *in vitro*.

a Higher thermogenic protein expression and adrenergic signaling in adipocytes with TonEBP haplo-deficiency. Immunoblots of PGC1α, p-CREB, Adrb3, UCP-1 and Hsc70 in primary adipocytes differentiated from SVF of WT (+/+) and TonEBP haplo-deficient mice (+/Δ). **b, c** TonEBP suppresses thermogenic gene expression in 3T3-L1 adipocytes. **(b)** 3T3-L1 cells transfected with siRNA were cultured in adipogenesis inducing medium (AIM) followed by treatment with isoproterenol (Iso) as indicated. **(c)** 3T3-L1 cells cultured in AIM were infected with adenovirus followed by treatment with Iso as indicated. All data are representative of four independent experiments. Data are presented as mean + s.d. **(b, c)**. # $p < 0.05$ vs. scr siRNA **(b)** or Ad-Empty **(c)**, * $p < 0.05$ vs. corresponding scr siRNA **(b)** or Ad-Empty **(c)**. **(b, c)** A 1-way ANOVA was used for comparisons between more than two conditions. Tukey's post-hoc test was used for multiple comparisons. AU, arbitrary unit.

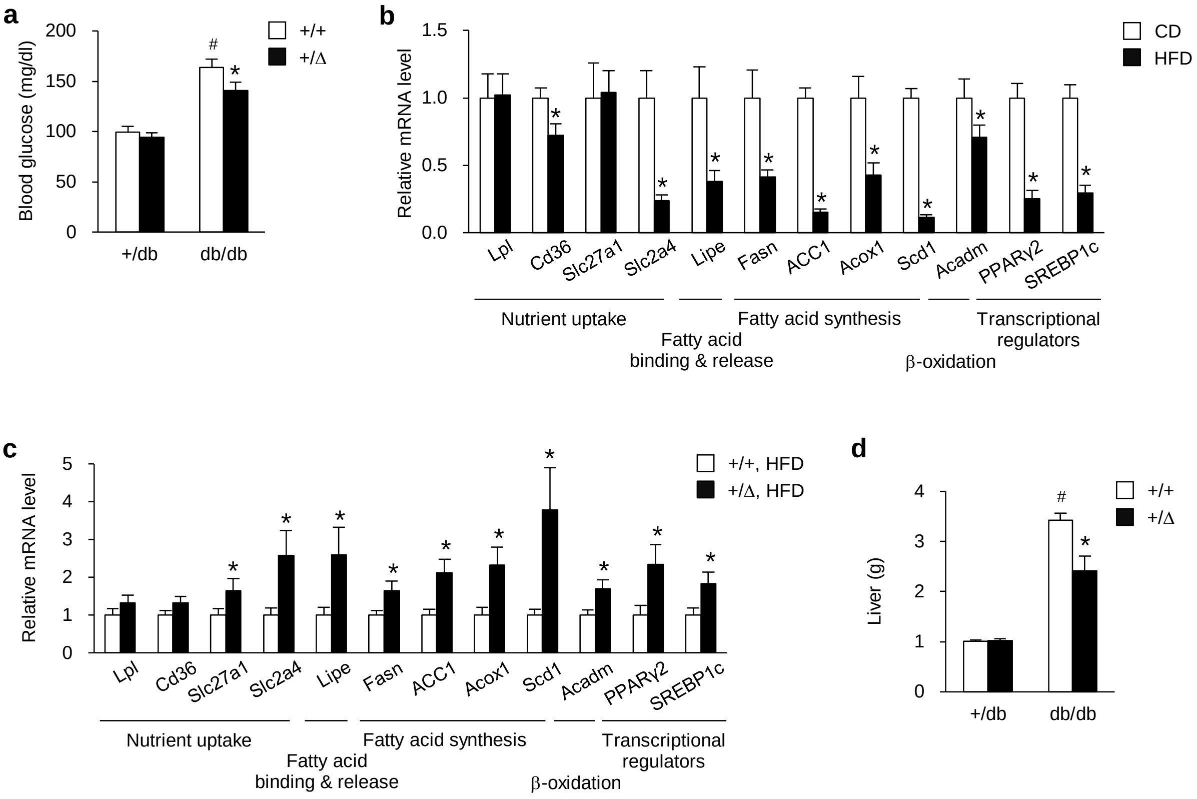

Supplementary Figure 5. Effects of TonEBP deficiency on metabolic dysfunction.

a TonEBP haplo-deficiency decreases fasting blood glucose level in db/db mice. Fasting blood glucose levels of animals shown in Supplementary Figure S2b and c (TonEBP +/+, *n* = 7; TonEBP +/Δ, *n* = 10). Data are representative of two independent experiments and presented as mean + s.e.m. # $p < 0.05$ vs. +/db, * $p < 0.05$ vs. +/+. **b, c** Suppressed lipid metabolism-related genes in response to HFD were restored by TonEBP haplo-deficiency. **(b)** mRNA level of genes involved in lipid metabolism in eWAT from C57BL/6J mice fed CD (*n* = 5) or HFD (*n* = 7). **(c)** mRNA level of genes involved in lipid metabolism in eWAT from HFD-fed animals (TonEBP +/+, *n* = 7; TonEBP +/Δ, *n* = 10). All data are representative of three independent experiments and presented as mean + s.e.m. * $p < 0.05$ vs. CD **(b)** or TonEBP +/+ **(c)**. **d** TonEBP haplo-deficiency decreases liver weight in db/db mice. Weight of liver from animals shown in Supplementary Figure 2b and c (TonEBP +/+, *n* = 7; TonEBP +/Δ, *n* = 10). All data are representative of four independent experiments and presented as mean + s.e.m. # $p < 0.05$ vs. +/db, * $p < 0.05$ vs. TonEBP +/+.

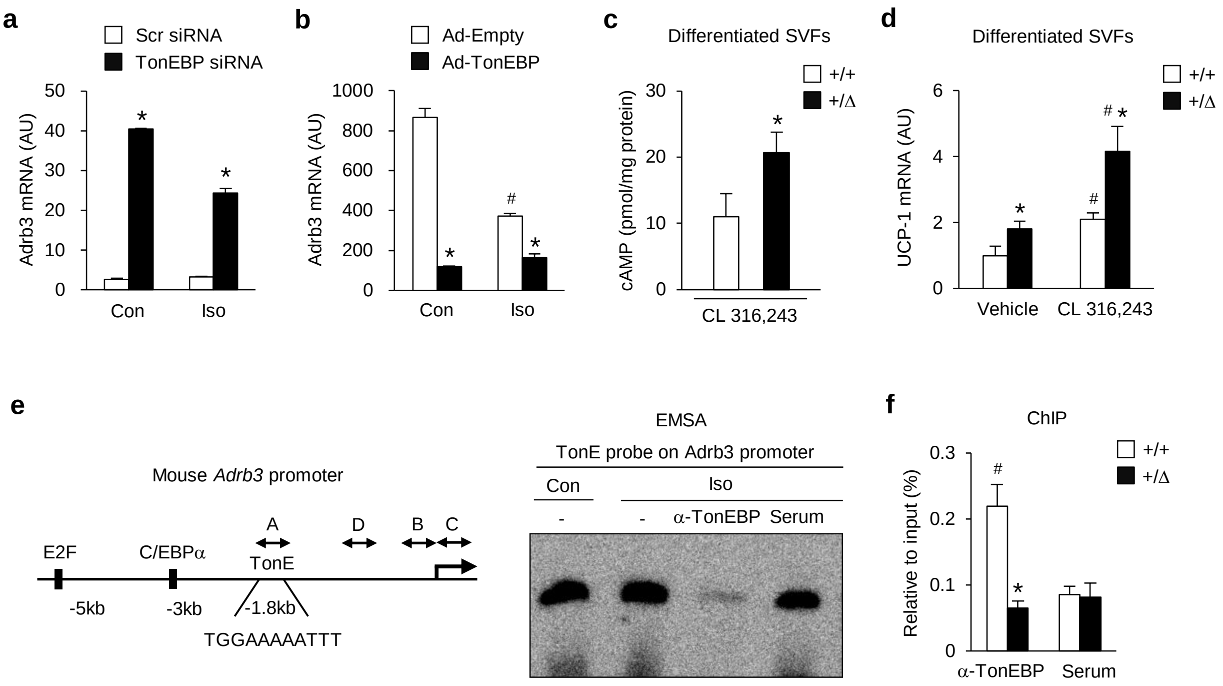

Supplementary Figure 6. Role of TonEBP on *Adrb3* gene expression.

a, b TonEBP suppresses *Adrb3* mRNA expression in 3T3-L1 adipocytes. *Adrb3* mRNA levels in 3T3-L1 adipocytes with siRNA-mediated TonEBP knockdown **(a)** or adenovirus-mediated TonEBP overexpression **(b)** were treated with isoproterenol (Iso) or not (Con) (n = 4). All data are representative of three independent experiments. **c** Adipocyte cAMP levels after treatment with an Adrb3 agonist were elevated in TonEBP haplo-deficiency. Intracellular cAMP levesl in CL 316,243 stimulated primary adipocytes differentiated from SVF of WT (+/+) and TonEBP haplo-deficient mice (+/Δ) (n = 3). **d** TonEBP haplo-deficiency enhances *UCP-1* mRNA expression. *UCP-1* mRNA levels in primary adipocytes from WT (+/+) and TonEBP haplo-deficient mice (+/Δ) after treatment with CL 316,243 (n = 3). Data are representative of two independent experiments. **e, f** TonEBP binds to the *Adrb3* promoter. **(e)** Nuclear extracts were prepared from 3T3-L1 cells treated with Iso or Con. EMSA was performed using a biotin labeled TonE probe. Where indicated, anti-TonEBP serum (α-TonEBP) or normal serum (Serum) were added for supershift of TonE-TonEBP complex. **(f)** ChIP assays targeting the B region in (**e**) for TonEBP on the *Adrb3* promoter (n = 4) using primary adipocytes from WT (+/+) and TonEBP haplo-deficient mice (+/Δ). Data are representative of two independent experiments. Data are presented as mean + s.d. **(a, b)** or s.e.m. **(c, d, f)**. # $p < 0.05$ vs. Con **(b)**, corresponding vehicle **(d)** or serum **(f)**, * $p < 0.05$ vs. corresponding scr siRNA **(a),** Ad-Empty **(b)**, corresponding +/+ **(c, d, f).**

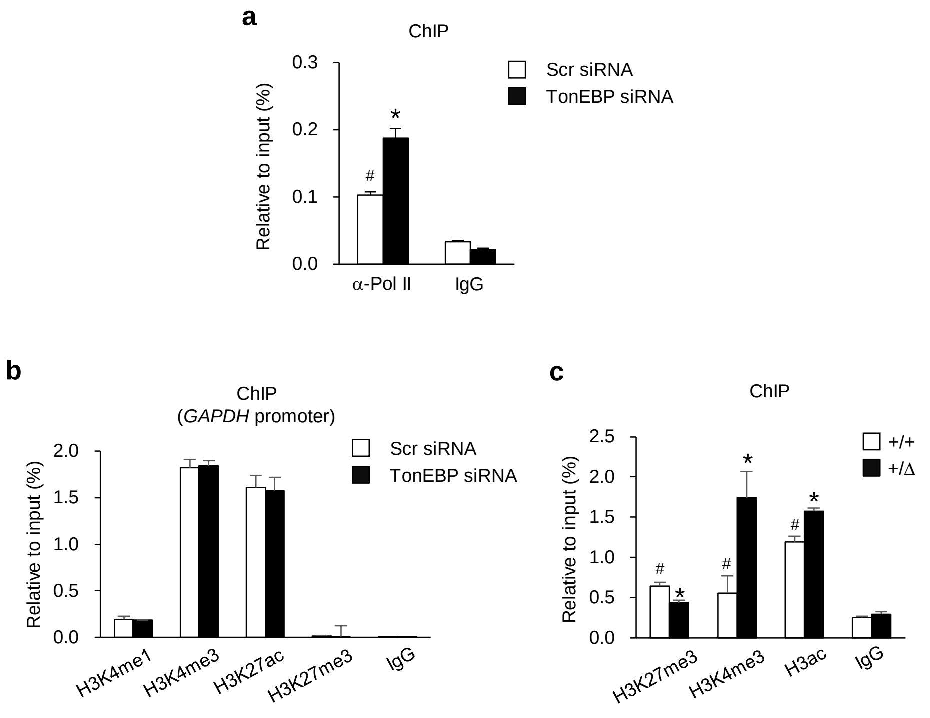

Supplementary Figure 7. Role of TonEBP on epigenetic changes in gene promoters.

a RNA polymerase II binding to the *Adrb3* promoter was elevated by TonEBP deficiency. ChIP assays targeting the B region in (Supplementary Figure 6e) for RNA polymerase II (Pol II) were performed using 3T3-L1 cells transfected with Scr and TonEBP siRNA. Data are representative of two independent experiments. **b** As a control, epigenetic changes in H3K4me1, H3K4me3, H3K27me3 and H3K27ac at the GA*PDH* promoter were performed using 3T3-L1 cells transfected with Scr and TonEBP siRNA. Data are representative of two independent experiments. **c** Epigenetic changes of the *Adrb3* promoter in response to TonEBP haplo-deficiency. ChIP assays for H3K27me3, H3K4me3 and H3ac on the B region in (Supplementary Figure 6e) of *Adrb3* promoter (n = 4) were performed in primary adipocytes from WT (+/+) and TonEBP haplo-deficient mice (+/Δ). Data presented as the mean + s.d. **(a, b)** or s.e.m. **(c)**. # $p < 0.05$ vs. corresponding IgG **(a-c)** , * $p < 0.05$ vs. corresponding scr siRNA **(a, b)**, or +/+ **(c).**

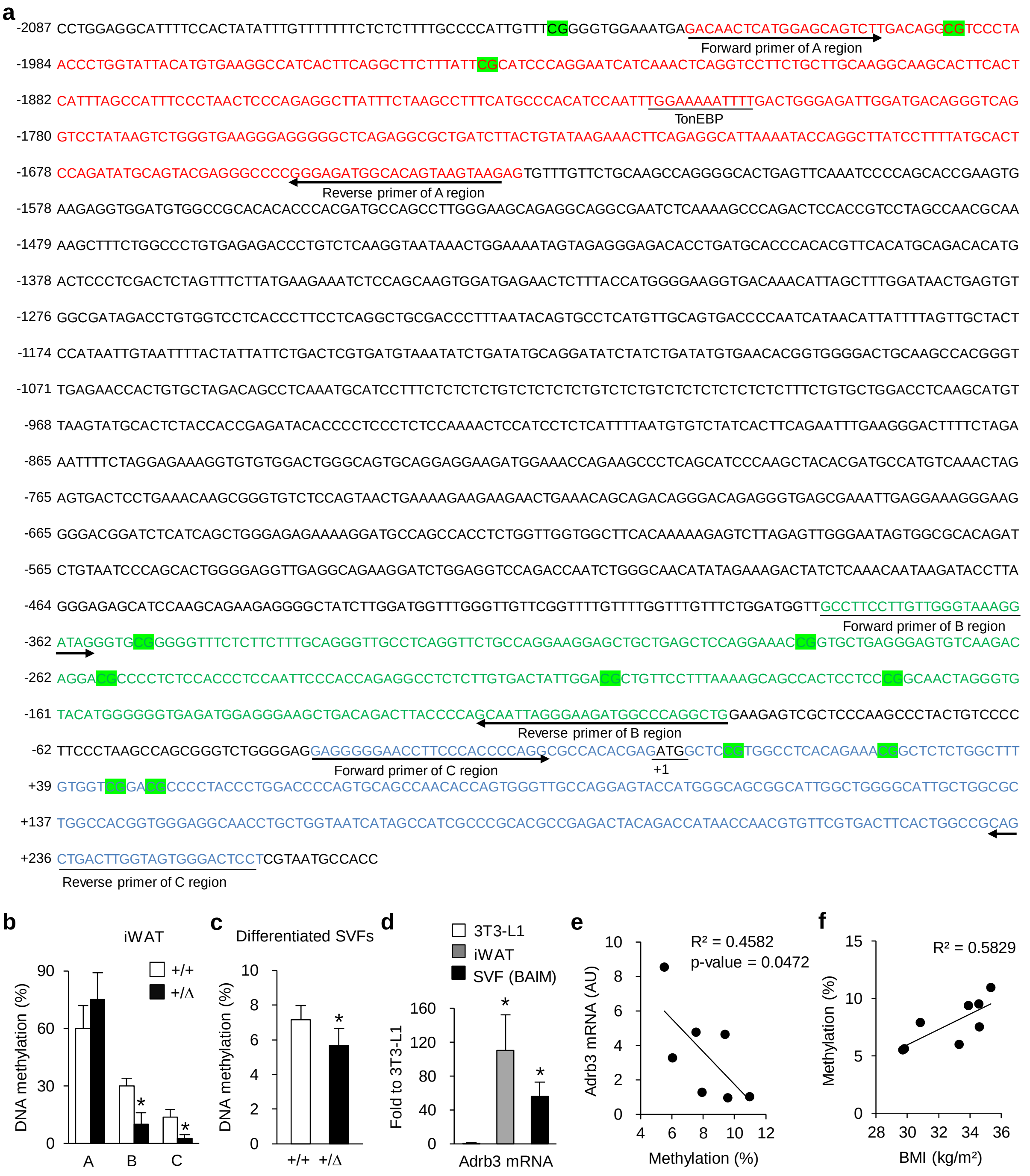

Supplementary Figure 8. Role of TonEBP in DNA methylation of *Adrb3* promoter.

a Nucleotide sequence of the mouse *Adrb3* promoter region. Nucleotides are numbered from the first codon (ATG). The 12 CpG sites targeted by bisulfite sequencing are marked with greed boxes. A, B, and C regions are indicated with distinct colors with PCR primers used. The TonEBP binding site (TonE) in A region is underlined. **b, c** DNA methylation of the *Adrb3* promoter was reduced by TonEBP deficiency. DNA methylation analyses were performed on the *Adrb3* promoter using bisulfite sequencing using iWAT (**b**; A, B, and C regions) and primary adipocytes differentiated from SVF (**c**, B region). Data are presented as the mean + s.e.m. * $p < 0.05$ vs. +/+. **d** *Adrb3* mRNA levels in 3T3-L1 cells (n=10), iWAT(n=6) and SVF (n=6). Mean + s.e.m. * $p < 0.05$ vs. 3T3-L1 cells. **e, f** *Adrb3* DNA methylation correlates with *Adrb3* mRNA expression and BMI. Correlation of DNA methylation levels of *Adrb3* promoter in human subcutaneous adipocytes with *Adrb3* mRNA expression **(e)** and BMI **(f)** (n = 7).

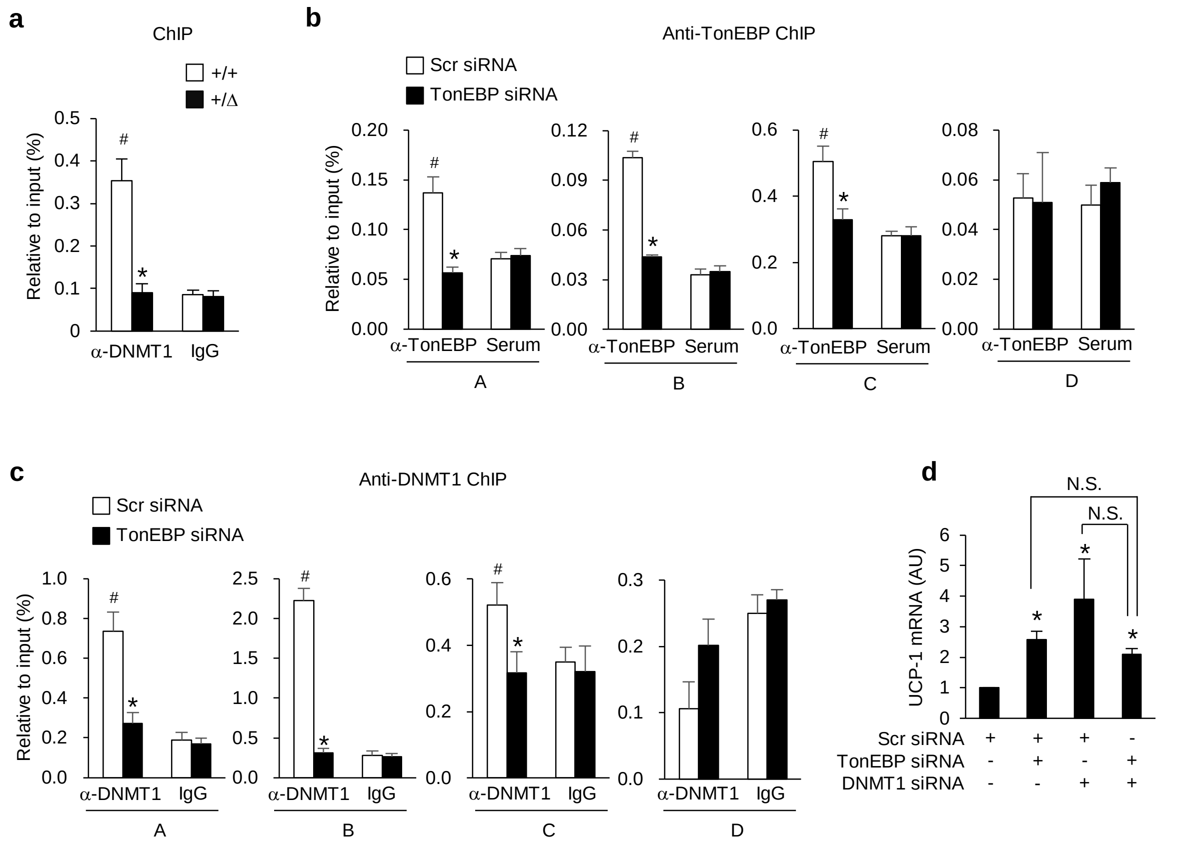

Supplementary Figure 9. Role of TonEBP in the recruitment of DNMT1 to the *Adrb3* promoter. **a** ChIP assays for DNMT1 on the *Adrb3* promoter (B region in Supplementary Figure 5e) (n = 4) using primary adipocytes from WT (+/+) and TonEBP haplo-deficient mice (+/Δ). **b, c** TonEBP and DNMT1 bind to the A, B and C regions but not D region, a negative control region (Supplementary Figure 5e) of the *Adrb3* promoter in a manner dependent on TonEBP. ChIP assays were performed for TonEBP **(b)** and DNMT1 **(c)** in 3T3-L1 cells transfected with scrambled (Scr) and TonEBP siRNA. **d** TonEBP and DNMT1 suppress *UCP-1* mRNA expression. *UCP-1* mRNA abundance was measured in 3T3-L1 adipocytes transfected with various siRNA's as indicated. **(a-d)** Data are representative of two independent experiments and presented as the mean + s.d. # $p < 0.05$ vs. corresponding IgG **(a, c)** or serum **(b)**. * $p < 0.05$ vs. +/+ **(a)** or Scr siRNA **(b-d)**. N.S., not significant **(d)**.

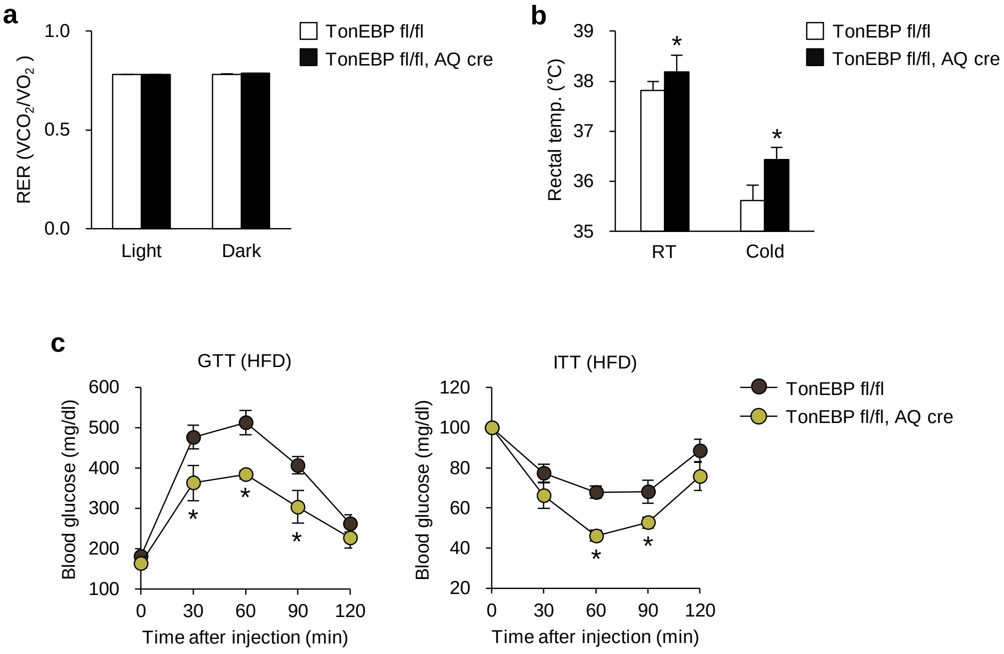

Supplementary Figure 10. Higher rectal temperature and insulin sensitivity in adipocyte-specific TonEBP deficiency.

a Respiratory exchange ratio (RER) was not affected by adipocyte-specific TonEBP deficiency. HFD-fed animals were analyzed by CLAMS to obtain RER (VCO_2/VO_2) (n = 4). Data are representative of three independent experiments and presented as mean + s.e.m. **b** Rectal temperature (temp.) measured in HFD-fed animals at room temperature (RT) and after exposure to 4°C (Cold) for 6 h (n = 7). **c** Glucose tolerance test (GTT, left) and insulin tolerance test (ITT, right) after 9 weeks on HFD. Data are presented as mean + s.e.m. * $p < 0.05$ vs. TonEBP +/+.

a

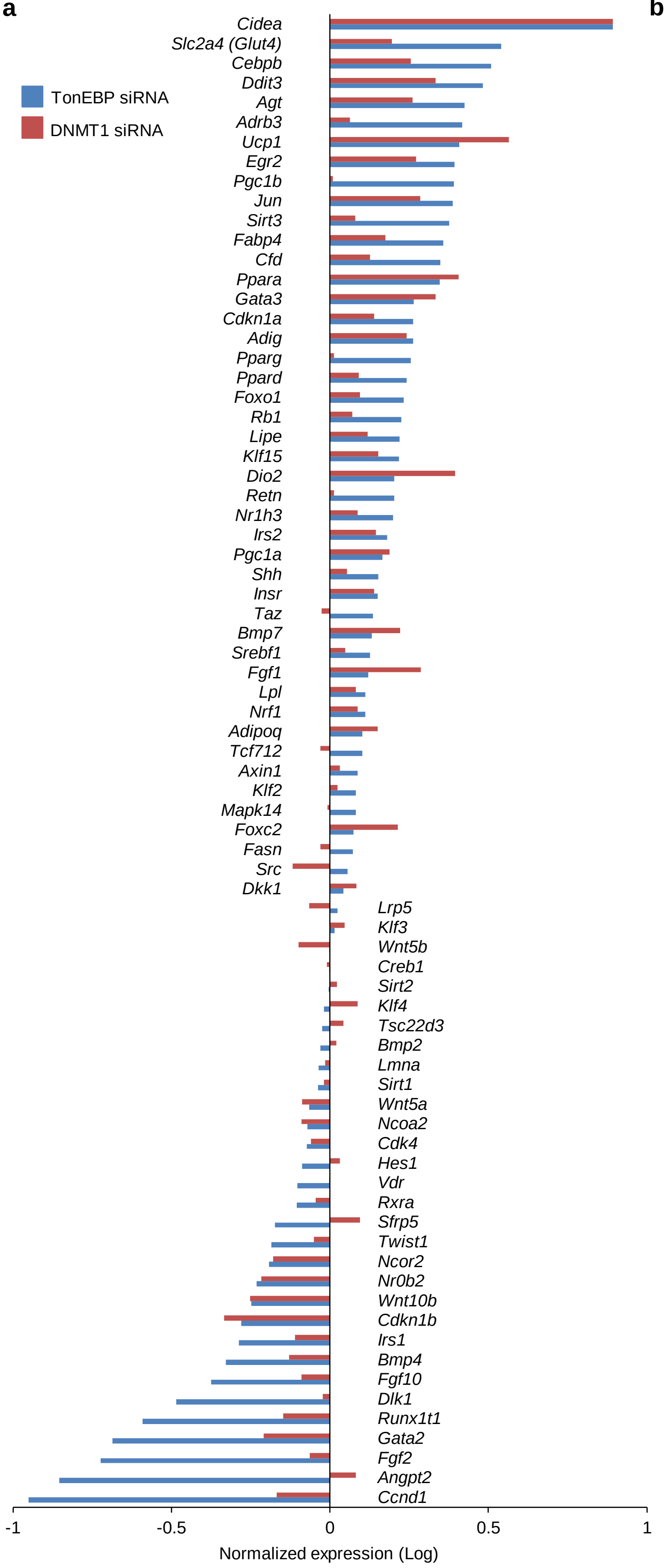

b

TonEBP siRNA \ DNMT1 siRNA	Up	Down	No change
Up	**31**	1	10
Down	0	**10**	10
No change	2	2	**10**

No.	Detail	Gene symbol	TonEBP siRNA	DNMT1 siRNA
1	Adipokine	Adig	up	up
2	Adipokine	Adipoq	up	up
3	Adipokine	Cfd	up	up
4	Adipokine	Retn	up	no change
5	Anti-adipogenesis	Cdkn1a	up	up
6	Anti-adipogenesis	Cdkn1b	down	down
7	Anti-adipogenesis	Ddit3	up	up
8	Anti-adipogenesis	Dlk1	down	no change
9	Anti-adipogenesis	Foxo1	up	up
10	Anti-adipogenesis	Hes1	down	no change
11	Anti-adipogenesis	Lrp5	no change	down
12	Anti-adipogenesis	Ncor2	down	down
13	Anti-adipogenesis	Runx1t1	down	down
14	Anti-adipogenesis	Shh	no change	no change
15	Anti-adipogenesis	Sirt1	no change	no change
16	Anti-adipogenesis	Sirt2	no change	no change
17	Anti-adipogenesis	Taz	up	no change
18	Anti-adipogenesis	Tcf712	up	no change
19	Anti-adipogenesis	Tsc22d3	down	no change
20	Anti-adipogenesis	Vdr	no change	no change
21	Anti-brown adipose tissue	Ncoa2	down	down
22	Anti-brown adipose tissue	Nr0b2	down	no change
23	Anti-brown adipose tissue	Nr1h3	up	up
24	Anti-brown adipose tissue	Rb1	up	up
25	Anti-brown adipose tissue	Twist1	down	down
26	Anti-brown adipose tissue	Wnt10b	down	down
27	Anti-white adipose tissue	Gata2	down	down
28	Anti-white adipose tissue	Gata3	up	up
29	Anti-white adipose tissue	Klf2	up	no change
30	Anti-white adipose tissue	Klf3	no change	no change
31	Hormone	Agt	up	up
32	Hormone	Angpt2	down	no change
33	Lipase	Lipe	up	up
34	Pro-adipogenesis	Axin1	up	no change
35	Pro-adipogenesis	Ccnd1	down	down
36	Pro-adipogenesis	Cdk4	down	no change
37	Pro-adipogenesis	Cebpb	up	up
38	Pro-adipogenesis	Dkk1	up	up
39	Pro-adipogenesis	Fabp4	up	up
40	Pro-adipogenesis	Fasn	up	down
41	Pro-adipogenesis	Fgf1	up	up
42	Pro-adipogenesis	Fgf2	down	no change
43	Pro-adipogenesis	Irs2	up	up
44	Pro-adipogenesis	Lmna	no change	no change
45	Pro-adipogenesis	Pparg	up	no change
46	Pro-adipogenesis	Rxra	down	no change
47	Pro-adipogenesis	Sfrp5	down	no change
48	Pro-adipogenesis	Slc2a4(glut4)	up	up
49	Pro-adipogenesis	Wnt5b	no change	no change
50	Pro-adipogenesis, lipase	Lpl	up	up
51	Pro-adipogensis	Jun	up	up
52	Pro-brown adipose tissue	Adrb3	up	up
53	Pro-brown adipose tissue	Bmp7	up	up
54	Pro-brown adipose tissue	Cidea	up	up
55	Pro-brown adipose tissue	Creb1	no change	no change
56	Pro-brown adipose tissue	Dio2	up	up
57	Pro-brown adipose tissue	Foxc2	no change	up
58	Pro-brown adipose tissue	Insr	up	up
59	Pro-brown adipose tissue	Irs1	down	down
60	Pro-brown adipose tissue	Mapk14	up	no change
61	Pro-brown adipose tissue	Nrf1	up	no change
62	Pro-brown adipose tissue	Pgc1a	up	up
63	Pro-brown adipose tissue	Pgc1b	up	no change
64	Pro-brown adipose tissue	Ppara	up	up
65	Pro-brown adipose tissue	Ppard	up	up
66	Pro-brown adipose tissue	Sirt3	up	up
67	Pro-brown adipose tissue	Src	no change	no change
68	Pro-brown adipose tissue	Ucp1	up	up
69	Pro-brown adipose tissue	Wnt5a	no change	down
70	Pro-white adipose tissue	Bmp2	no change	no change
71	Pro-white adipose tissue	Bmp4	down	no change
72	Pro-white adipose tissue	Egr2	up	up
73	Pro-white adipose tissue	Fgf10	down	down
74	Pro-white adipose tissue	Klf15	up	up
75	Pro-white adipose tissue	Klf4	no change	up
76	Pro-white adipose tissue	Srebf1	up	no change

Supplementary Figure 11. Role of TonEBP and DNMT1 in the regulation of adipocyte physiology-related genes.

a PCR array (Qiagen) data on TonEBP or DNMT1 knockdowned 3T3-L1 adipocytes. Data are representative of three independent experiments. **b** The number (top) and list (bottom) of genes regulated by TonEBP or DNMT1 on PCR array.

Fig. 1b

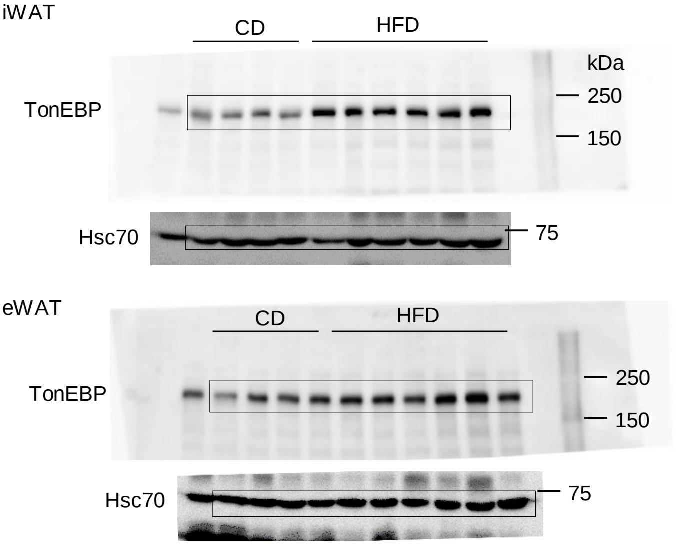

Fig. 1e

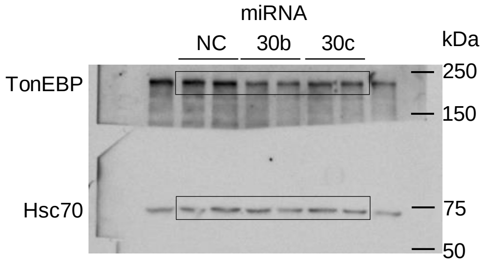

Fig. 2h

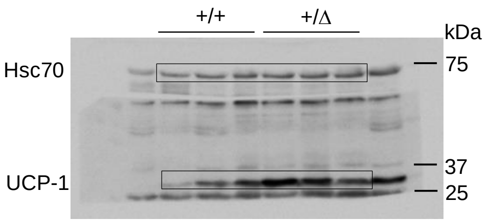

Fig. 3d

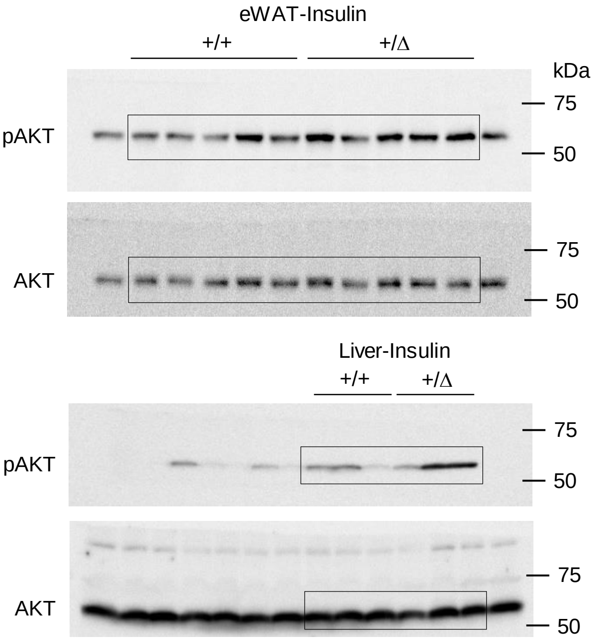

Supplementary Figure 12. Uncropped blots of Western blots in Figures 1-3. Original uncropped blots corresponding to Western blots in Figures 1-3 in the manuscript.

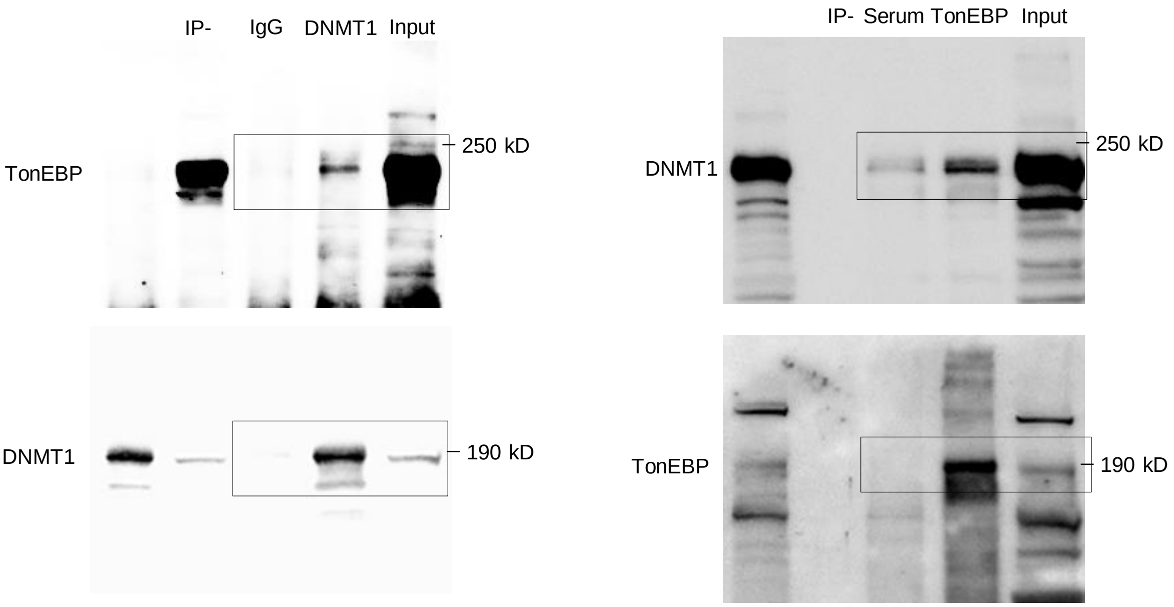

Supplementary Figure 13. Uncropped blots of Western blots in Figure 4. Original uncropped blots corresponding to Western blotting of immunoprecipitated proteins in Figure 4n in the manuscript.
