# Supplementary material for: TonEBP/NFAT5 promotes obesity and insulin resistance by epigenetic suppression of white adipose tissue beiging
Source: Nat Commun. 2019 Aug 6;10:3536. doi: 10.1038/s41467-019-11302-w (PMC6684655; doi:10.1038/s41467-019-11302-w)
Supplement: Supplementary file 3 — Reporting Summary [file 41467_2019_11302_MOESM3_ESM.pdf]

nature research

Corresponding author(s): DBPR: NCOMMS-17-29248D

Last updated by author(s): Jun 16, 2019

Reporting Summary

Nature Research wishes to improve the reproducibility of the work that we publish. This form provides structure for consistency and transparency in reporting. For further information on Nature Research policies, see Authors & Referees and the Editorial Policy Checklist.

Statistics

For all statistical analyses, confirm that the following items are present in the figure legend, table legend, main text, or Methods section.

n/a	Confirmed	
☐	☒	The exact sample size (*n*) for each experimental group/condition, given as a discrete number and unit of measurement
☐	☒	A statement on whether measurements were taken from distinct samples or whether the same sample was measured repeatedly
☐	☒	The statistical test(s) used AND whether they are one- or two-sided<br>*Only common tests should be described solely by name; describe more complex techniques in the Methods section.*
☒	☐	A description of all covariates tested
☐	☒	A description of any assumptions or corrections, such as tests of normality and adjustment for multiple comparisons
☐	☒	A full description of the statistical parameters including central tendency (e.g. means) or other basic estimates (e.g. regression coefficient) AND variation (e.g. standard deviation) or associated estimates of uncertainty (e.g. confidence intervals)
☐	☒	For null hypothesis testing, the test statistic (e.g. *F*, *t*, *r*) with confidence intervals, effect sizes, degrees of freedom and *P* value noted<br>*Give P values as exact values whenever suitable.*
☒	☐	For Bayesian analysis, information on the choice of priors and Markov chain Monte Carlo settings
☒	☐	For hierarchical and complex designs, identification of the appropriate level for tests and full reporting of outcomes
☐	☒	Estimates of effect sizes (e.g. Cohen's *d*, Pearson's *r*), indicating how they were calculated

Our web collection on statistics for biologists contains articles on many of the points above.

Software and code

Policy information about availability of computer code

Data collection	No software was used
Data analysis	Microsoft Excel 2013, Quantity one, Prism 8 and ImageJ 1.52e were used for data analysis.

For manuscripts utilizing custom algorithms or software that are central to the research but not yet described in published literature, software must be made available to editors/reviewers. We strongly encourage code deposition in a community repository (e.g. GitHub). See the Nature Research guidelines for submitting code & software for further information.

Data

Policy information about availability of data

All manuscripts must include a data availability statement. This statement should provide the following information, where applicable:

- Accession codes, unique identifiers, or web links for publicly available datasets
- A list of figures that have associated raw data
- A description of any restrictions on data availability

Data supporting the findings of this study are available from the corresponding authors upon reasonable request. The source data underlying Figures 1-6 are provided as a Source Data file.

Field-specific reporting

Please select the one below that is the best fit for your research. If you are not sure, read the appropriate sections before making your selection.

☒ Life sciences ☐ Behavioural & social sciences ☐ Ecological, evolutionary & environmental sciences

Life sciences study design

All studies must disclose on these points even when the disclosure is negative.

Sample size	The sample size was large enough to determine statistically significant effects and determined based on other studies with similar methodologies.
Data exclusions	No data were excluded from the analyses.
Replication	Experiments were repeated independently at least 3 times with at least 3 replicates.
Randomization	The groups that receive different experimental treatments are determined randomly.
Blinding	Blind test was performed for data collection and analysis.

Reporting for specific materials, systems and methods

We require information from authors about some types of materials, experimental systems and methods used in many studies. Here, indicate whether each material, system or method listed is relevant to your study. If you are not sure if a list item applies to your research, read the appropriate section before selecting a response.

Materials & experimental systems

n/a	Involved in the study	
☐	☒	Antibodies
☐	☒	Eukaryotic cell lines
☐	☐	Palaeontology
☐	☒	Animals and other organisms
☐	☒	Human research participants
☐	☐	Clinical data

Methods

n/a	Involved in the study	
☒	☐	ChIP-seq
☒	☐	Flow cytometry
☒	☐	MRI-based neuroimaging

Antibodies

Antibodies used	Anti-TonEBP serum (Immunoblotting, ChIP; manufactured in our lab (Miyakawa et al., PNAS, 96:2538-42, 1999)), Anti-DNMT1 antibody (Immunoblotting, ChIP; Abcam, ab188453 [EPR18453], GR314973-4), Anti-UCP-1 antibody (Immunoblotting, IHC; Milipore, AB1426), Anti-HSC70 antibody (Immunoblotting, Rockland, 200-301-A28, 35378), Anti-AKT antibody (Immunoblotting, Cell signaling, 9272), Anti-pAKT antibody (Immunoblotting, Cell signaling, 9271S, 13), Anti-Adrb3 antibody (IHC; Abcam, ab59685, GR3558-20), Anti-F4/80 antibody (IHC; Abcam, ab6640 [CI:A3-11], GR3558-20), Anti-PolII antibody (ChIP; Abcam, ab817, GR3216482-3), Anti-H3K4me1 antibody (ChIP; Abcam, ab8895, GR3235544-1), Anti-H3K4me3 antibody (ChIP; Abcam, ab8580, GR3197347-1), Anti-H3K27me3 antibody (ChIP; Abcam, ab6002, GR3232152-1), Anti-H3K27ac antibody (ChIP; Abcam, ab4729, GR3216173-1), Anti-H3ac antibody (ChIP; Millipore, 06-299), normal rabbit antibody (ChIP; Abcam, ab171870, GR3228514-3)
Validation	Anti-TonEBP serum was validated in our studies (Miyakawa et al., PNAS, 96:2538-42, 1999). The validation statements of commercial antibodies were described on the manufacturer's website.

Eukaryotic cell lines

Policy information about cell lines

Cell line source(s)	3T3-L1 (CR-173) and HEK293 (CRL-1573) were obtained from ATCC.
Authentication	3T3-L1 cells were authenticated by lipid accumulation on adipocyte differentiation and and cell morphology by microscopy. HEK293 cells were authenticated by expression of vitronectin and cell morphology.
Mycoplasma contamination	Cells were negative for mycoplasmal contamination by DAPI staining.
Commonly misidentified lines (See ICLAC register)	No commonly misidentified cell lines were used.

Palaeontology

Specimen provenance	*Provide provenance information for specimens and describe permits that were obtained for the work (including the name of the issuing authority, the date of issue, and any identifying information).*

Specimen deposition	*Indicate where the specimens have been deposited to permit free access by other researchers.*
Dating methods	*If new dates are provided, describe how they were obtained (e.g. collection, storage, sample pretreatment and measurement), where they were obtained (i.e. lab name), the calibration program and the protocol for quality assurance OR state that no new dates are provided.*

☐ Tick this box to confirm that the raw and calibrated dates are available in the paper or in Supplementary Information.

Animals and other organisms

Policy information about studies involving animals; ARRIVE guidelines recommended for reporting animal research

Laboratory animals	8 weeks old male C57BL/6 mice (wild type, TonEBP haplodeficiency, TonEBP floxed mutant, adipocyte-specific TonEBP knockout and db/db mice)
Wild animals	*Provide details on animals observed in or captured in the field; report species, sex and age where possible. Describe how animals were caught and transported and what happened to captive animals after the study (if killed, explain why and describe method; if released, say where and when) OR state that the study did not involve wild animals.*
Field-collected samples	*For laboratory work with field-collected samples, describe all relevant parameters such as housing, maintenance, temperature, photoperiod and end-of-experiment protocol OR state that the study did not involve samples collected from the field.*
Ethics oversight	All the methods involving live mice were carried out in accordance with the approved guidelines. All experimental protocols were approved by Institutional Animal Care and Use Committee of the Ulsan National Institute of Science and Technology (UNISTACUC-12-15-A)

Note that full information on the approval of the study protocol must also be provided in the manuscript.

Human research participants

Policy information about studies involving human research participants

Population characteristics	Adipocyte RNA of subcutaneous abdominal adipose tissues were obtained from 15 Saudi Arabian subjects.
Recruitment	The procedures were carried out in accordance with the approved protocol, with written informed consent obtained from all subjects. Further information and requests for resources and raw data should be directed and will be fulfilled by the Contact: A. A. Alfadda, aalfadda@ksu.edu.sa.
Ethics oversight	The local ethics committee of the College of Medicine, King Saud University approved experimental design and adipose tissue samples collection (approval code 07-602).

Note that full information on the approval of the study protocol must also be provided in the manuscript.

Clinical data

Policy information about clinical studies

All manuscripts should comply with the ICMJE guidelines for publication of clinical research and a completed CONSORT checklist must be included with all submissions.

Clinical trial registration	*Provide the trial registration number from ClinicalTrials.gov or an equivalent agency.*
Study protocol	*Note where the full trial protocol can be accessed OR if not available, explain why.*
Data collection	*Describe the settings and locales of data collection, noting the time periods of recruitment and data collection.*
Outcomes	*Describe how you pre-defined primary and secondary outcome measures and how you assessed these measures.*
